# Supplementary material for: Autoregulation of RPL7B by inhibition of a structural splicing enhancer
Source: Nucleic Acids Res. 2025 Jul 31;53(14):gkaf739. doi: 10.1093/nar/gkaf739 (PMC12311795; doi:10.1093/nar/gkaf739)
Supplement: gkaf739_Supplemental_Files [file gkaf739_supplemental_files.zip › supplemental figures and table.pdf]

Figure S1 Sequences of all the genes and proteins expressed in this work

>Rpl7a cre-less DNA (with C-terminal linker+mCherry in lowercase)
ATGGCTGCGGAGAAGATTTTGACTCCAGAATCCCAATTGAAGAAATCCAAGGCCCAACAAAAGACAGCTGAACAAGTTGC
TGCTGAAAGAGCTGCCAGAAAGGCCGCTAACAAGGAAAAGCGTGCTATCATCTTGGAAAGAAACGCTGCTTACCAAAAGG
AATACGAAACAGCTGAAAGAAACATTATCCAAGCTAAGAGAGACGCTAAGGCTGCTGGTTCTTACTATGTTGAAGCTCAG
CACAAACTAGTTTTCGTCGTTCGTATTAAGGGTATCAACAAGATCCCACCAAAGCCTAGAAAGGTCTTGCAATTGTTGAG
ACTAACTAGAATTAACTCGGGTACTTTCGTTAAGGTCACTAAAGCTACCTTGGAATTGTTGAAGCTAATCGAACCATACG
TCGCCTACGGTTACCCATCTTACTCCACCATCAGACAATTGGTTTACAAGAGAGGTTTCGGTAAGATTAACAAGCAAAGA
GTCCCATTGTCTGATAACGCCATCATCGAAGCTAACTTGGGTAAGTACGGTATTTTGTCTATCGATGACTTGATCCACGA
AATCATTACTGTCGGTCCACACTTCAAGCAAGCTAACAACTTCTTGTGGCCATTTAAGTTGTCTAACCCATCCGGTGGTT
GGGGTGTTCCACGTAAGTTCAAGCACTTCATCCAAGGTGGTTCCTTCGGTAACAGAGAAGAATTTATTAACAAGTTGGTC
AAGTCTATGAAT
gtgagcaagggcgaggagcctagggtgagcaagggcgaggaggataacatggccatcatcaaggagttcatgagattcaa
ggtgcacatggagggctccgtgaacggccacgagttcgagatcgagggcgagggcgagggcagaccctacgagggcaccc
agaccgccaagctgaaggtgaccaagggtggcccctgcccttcgcctgggacatcctgtcccctcagttcatgtacggc
tccaaggcctacgtgaagcaccccgccgacatccccgactacttgaagctgtccttccccgagggcttcaagtgggagag
agtgatgaacttcgaggacggcggcgtggtgaccgtgacccaggactcctccctgcaggacggcgagttcatctacaagg
tgaagttgagaggcaccaacttcccctccgacggccccgtaatgcagaagaagaccatgggctgggaggcctcctccgag
agaatgtaccccgaggacggcgccctgaagggcgagatcaagcagaggctgaagctgaaggacggcggccactacgacgc
tgaggtcaagaccacctacaaggccaagaagcccgtgcagctgcccggcgcctacaacgtcaacatcaagttggacatca
cctcccacaacgaggactacaccattgtggaacaatatgaaagagctgaaggtagacattctactggtggtatggatgaa
ttgtacaaataa

>Rpl7b cre-less DNA (with C-terminal linker+mCherry in lowercase)
ATGTCCACTGAAAAGATTTTGACCCCAGAATCCCAATTGAAAAAGACTAAGGCCCAACAAAAGACTGCTGAACAAATCGC
CGCTGAAAGAGCTGCTAGAAAGGCTGCTAATAAGGAAAAGAGAGCAATCATCTTGGAGAGAAATGCTGCCTACCAAAAAG
AATACGAAACAGCCGAAAGAAACATTATCCAAGCTAAGAGAGACGCTAAAGCTGCTGGTTCTTACTACGTTGAAGCCCAA
CACAAATTGGTTTTCGTCGTTCGTATCAAGGGTATCAACAAGATCCCACCAAAGCCAAGAAAGGTCTTGCAATTGTTGAG
ATTGACTAGAATTAACTCCGGTACTTTCGTTAAGGTCACTAAGGCTACATTGGAATTGTTGAAGTTGATCGAACCTTACG
TCGCTTACGGTTACCCATCTTATTCCACTATCAGACAATTGGTTTACAAGCGTGGTTTCGGTAAAATTAACAAGCAACGT
GTCCCATTGTCTGATAACGCAATTATTGAAGCTAACTTGGGTAAGTACGGTATTTTGTCTATCGATGACCTAATCCACGA
AATTATCACTGTCGGTCCACATTTCAAACAAGCTAACAACTTCCTATGGCCATTCAAGTTGTCTAACCCTTCCGGTGGTT
GGGGTGTTCCAAGAAAGTTTAAGCACTTCATTCAAGGTGGTTCCTTCGGTAACAGAGAAGAATTCATTAACAAGCTAGTT
AAGGCTATGAAC
gtgagcaagggcgaggagcctagggtgagcaagggcgaggaggataacatggccatcatcaaggagttcatgagattcaa
ggtgcacatggagggctccgtgaacggccacgagttcgagatcgagggcgagggcgagggcagaccctacgagggcaccc
agaccgccaagctgaaggtgaccaagggtggcccctgcccttcgcctgggacatcctgtcccctcagttcatgtacggc
tccaaggcctacgtgaagcaccccgccgacatccccgactacttgaagctgtccttccccgagggcttcaagtgggagag
agtgatgaacttcgaggacggcggcgtggtgaccgtgacccaggactcctccctgcaggacggcgagttcatctacaagg
tgaagttgagaggcaccaacttcccctccgacggccccgtaatgcagaagaagaccatgggctgggaggcctcctccgag
agaatgtaccccgaggacggcgccctgaagggcgagatcaagcagaggctgaagctgaaggacggcggccactacgacgc
tgaggtcaagaccacctacaaggccaagaagcccgtgcagctgcccggcgcctacaacgtcaacatcaagttggacatca
cctcccacaacgaggactacaccattgtggaacaatatgaaagagctgaaggtagacattctactggtggtatggatgaa
ttgtacaaataa

>Rpl7a cre-less protein (with C-terminal linker+mCherry in lowercase)
MAAEKILTPESQLKKSKAQQKTAEQVAAERAARKAANKEKRAIILERNAAYQKEYETAERNIIQAKRDAKAAGSYYVEAQ
HKLVFVVRIKGINKIPPKPRKVLQLLRLTRINSGTFVKVTKATLELLKLIEPYVAYGYPSYSTIRQLVYKRGFGKINKQR
VPLSDNAIIEANLGKYGILSIDDLIHEIITVGPHFKQANNFLWPFKLSNPSGGWGVPRKFKHFIQGGSFGNREEFINKLV
KSMN
vskgeeprvskgeednmaiikefmrfkvhmegsvnghefeiegegegrpyegtqtaklkvtkggplpfawdilspqfmyg
skayvkhpadipdylklsfpegfkwervmnfedggvvtvtqdsslqdgefiykvklrgtnfpsdgpvmqkktmgweasse
rmypedgalkgeikqrlklkdgghydaevkttykakkpvqlpgaynvniklditshnedytiveqyeraegrhstggmde
lyk*

>Rpl7b cre-less protein (with C-terminal linker+mCherry in lowercase)
MSTEKILTPESQLKKTKAQQKTAEQIAAERAARKAANKEKRAIILERNAAYQKEYETAERNIIQAKRDAKAAGSYYVEAQ
HKLVFVVRIKGINKIPPKPRKVLQLLRLTRINSGTFVKVTKATLELLKLIEPYVAYGYPSYSTIRQLVYKRGFGKINKQR
VPLSDNAIIEANLGKYGILSIDDLIHEIITVGPHFKQANNFLWPFKLSNPSGGWGVPRKFKHFIQGGSFGNREEFINKLV
KAMN
vskgeeprvskgeednmaiikefmrfkvhmegsvnghefeiegegegrpyegtqtaklkvtkggplpfawdilspqfmyg

skayvkhpadipdylklsfpegfkwervmnfedggvvtvtqdsslqdgefiykvklrgtnfpsdgpvmqkktmgweasse
rmypedgalkgeikqrlklkdgghydaevkttykakkpvqlpgaynvniklditshnedytiveqyeraegrhstggmde
lyk*

>Alignment between Rpl7a and Rpl7b protein sequences

```
Rpl7a   MAAEKILTPESQLKKSKAQQKTAEQVAAERAARKAANKEKRAIILERNAAYQKEYETAERNIIQAKRDAKAA
Rpl7b   .ST............T.........I..............................................
Rpl7a   GSYYVEAQHKLVFVVRIKGINKIPPKPRKVLQLLRLTRINSGTFVKVTKATLELLKLIEPYVAYGYPSYSTI
Rpl7b   ........................................................................
Rpl7a   RQLVYKRGFGKINKQRVPLSDNAIIEANLGKYGILSIDDLIHEIITVGPHFKQANNFLWPFKLSNPSGGWGV
Rpl7b   ........................................................................
Rpl7a   PRKFKHFIQGGSFGNREEFINKLVKSMN
Rpl7b   .........................A..
```

>Rpl7a-zz DNA (from the moveable ORF collection)
ATGGCCGCTGAGTATGTATACGAAATCGTCTATTTTATTACTATTTCCTAAAGTATCCAGAGAAGTATACTAGTTTCCGC
AAGTTACGTTGAGAAAAAGGAGCAATTCTGCTCTAATAGCATTTGCACTCACACTTACATTTATCTTCAACTTGTGACTA
TTCACAAGATGATTTTCCGTAGGGGATTACCTTCACAGGGTCAAAGACAATTTTAGTTGATGAATTTGAGGAAAAATTTG
AAATATCTTGATTGTTGCCAAAAATTTCATCGCCGACCTTGATTAGAATATTTTCTCAAATTTTCATTTCCTAAGTGTCT
CTGTTTCACTCCCTTCATTCGAATGAAAAGTGGTGTTTTCAATTCGATGTAACTTCGTAGAAAAAAGTTTTACTTGATGG
TCATACAAACCTAATTCATTCTATTTTCGGTTATGCTAACAAAAATATTTTATTATTTGTATTATTACAGAAAAATCTTG
ACCCCAGAATCTCAGTTGAAGAAGTCTAAGGCTCAACAAAAGACTGCTGAACAAGTCGCTGCTGAAAGAGCTGCTCGTAA
GGCTGTATGTTAAACTTTTGCTTACATTTATATTTTATGCTGCAACACATTTTCTTACTGGGATATATGTGAGTTCACGT
GCGTCGCCAATTTTGATAAGTATCATTCGAACAGTTGCAATATTCTCAACACCTGTGGTATCATTAGGCTAATGGACTAT
TTGTTACGTTCTGTTTGTTTTCTCATTTTATGATGAAAATCCATTGTATCCCAATACATATTCGACTAGTCATTGACGAT
GCTGTCGTAACTTATCACCATCTTTCGACTGATTATAAGAAAAGAAATAGAAAGTAAAATAGACCAGAGAGTTAAGGTAT
TAAGGTTACAATTATTGCCGTGTTTCCAGTGTCAGAAAAACCATTGAGTGATAATGCCGTGTAACAATTTTTAGCATAAA
ATACTGTGTTTTCCATCTGGTATTTTTTTACTAACATCAATTTACTGTTTTTTACTTTTTATTTTCATTTAGGCTAACAA
GGAAAAGAGAGCCATTATTTTGGAAAGAAACGCCGCTTACCAAAAGGAATACGAAACTGCTGAAAGAAACATCATTCAAG
CTAAGCGTGATGCCAAGGCTGCTGGTTCCTACTACGTCGAAGCTCAACACAAGTTGGTCTTCGTTGTCAGAATCAAGGGT
ATTAACAAGATCCCACCTAAGCCAAGAAAGGTTCTACAATTGCTAAGATTGACAAGAATCAACTCTGGTACATTCGTCAA
AGTTACCAAGGCTACTTTGGAACTATTGAAGTTGATTGAACCATACGTTGCTTACGGTTACCCATCGTACTCTACTATTA
GACAATTGGTCTACAAGAGAGGTTTCGGTAAGATCAACAAGCAAAGAGTTCCATTGTCCGACAATGCTATCATCGAAGCC
AACTTGGGTAAGTATGGTATCTTGTCCATTGACGATTTGATTCACGAAATCATCACTGTTGGTCCACACTTCAAGCAAGC
TAACAACTTTTTGTGGCCATTCAAGTTGTCCAACCCATCTGGTGGTTGGGGTGTCCCAAGAAAGTTCAAGCACTTTATCC
AAGGTGGTTCTTTCGGTAACCGTGAAGAATTCATCAACAAATTGGTTAAGTCCATGAACAACCCAGCTTTCTTGTACAAA
GTGGTTGATGTGTCTAGACATCATCATCACCATCATGGTAGAATCTTTTATCCATACGATGTTCCTGATTATGCTGGTTT
AGAAGTTCTTTTTCAAGGTCCTGGACCATCGGCCGTGGACAACAAATTCAACAAAGAACAACAAAACGCGTTCTATGAGA
TCTTACATTTACCTAACTTAAACGAAGAACAACGAAACGCCTTCATCCAAAGTTTAAAAGATGACCCAAGCCAAAGCGCT
AACCTTTTAGCAGAAGCTAAAAAGCTAAATGATGCTCAGGCGCCGAAAGTAGACAACAAATTCAACAAAGAACAACAAAA
CGCGTTCTATGAGATCTTACATTTACCTAACTTAAACGAAGAACAACGAAACGCCTTCATCCAAAGTTTAAAAGATGACC
CAAGCCAAAGCGCTAACCTTTTAGCAGAAGCTAAAAAGCTAAATGATGCTCAGGCGCCGAAAGTAGACCCGAATCATCAG

>Rpl7a-zz protein (from the moveable ORF collection)
MAAEKILTPESQLKKSKAQQKTAEQVAAERAARKAANKEKRAIILERNAAYQKEYETAERNIIQAKRDAKAAGSYYVEAQ
HKLVFVVRIKGINKIPPKPRKVLQLLRLTRINSGTFVKVTKATLELLKLIEPYVAYGYPSYSTIRQLVYKRGFGKINKQR
VPLSDNAIIEANLGKYGILSIDDLIHEIITVGPHFKQANNFLWPFKLSNPSGGWGVPRKFKHFIQGGSFGNREEFINKLV
KSMNNPAFLYKVVDVSRHHHHHHGRIFYPYDVPDYAGLEVLFQGPGPSAVDNKFNKEQQNAFYEILHLPNLNEEQRNAFI
QSLKDDPSQSANLLAEAKKLNDAQAPKVDNKFNKEQQNAFYEILHLPNLNEEQRNAFIQSLKDDPSQSANLLAEAKKLND
AQAPKVDPNHQ

Figure S2 mCherry fluorescence signal for all experiments

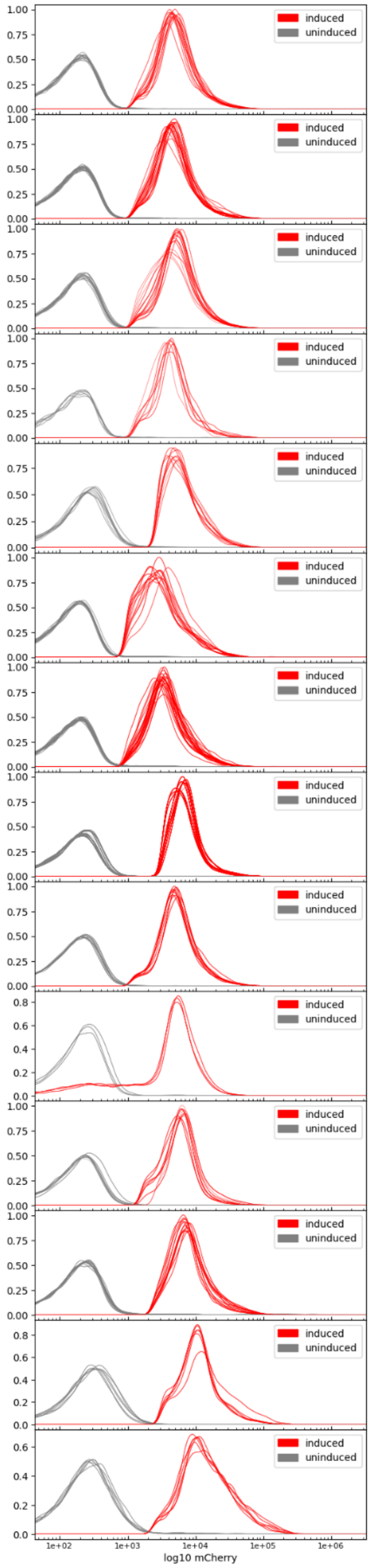

CREless protein: RPL7A
experiment: v1

CREless protein: RPL7A
experiment: v2

CREless protein: RPL7A
experiment: v3

CREless protein: RPL7A
experiment: v4

CREless protein: RPL7A
experiment: v5

CREless protein: RPL7A
experiment: v6

CREless protein: RPL7A
experiment: v7v8

CREless protein: RPL7A
experiment: v9

CREless protein: RPL7A
experiment: test-introns

CREless protein: RPL7A
experiment: test-introns-7a

CREless protein: RPL7A
experiment: test-7A-introns-with-both-CREless-proteins

CREless protein: RPL7A
experiment: test_rpl7b_creless

CREless protein: RPL7B
experiment: test-7A-introns-with-both-CREless-proteins

CREless protein: RPL7B
experiment: test_rpl7b_creless

Figure S3

Fluorescence of GFP with intron 1 of *RPL7A* (top two panels) and *RPL7B* (bottom two panels) with induced proteins Rpl7a (panels 1 and 3) and Rpl7b (panels 2 and 4)

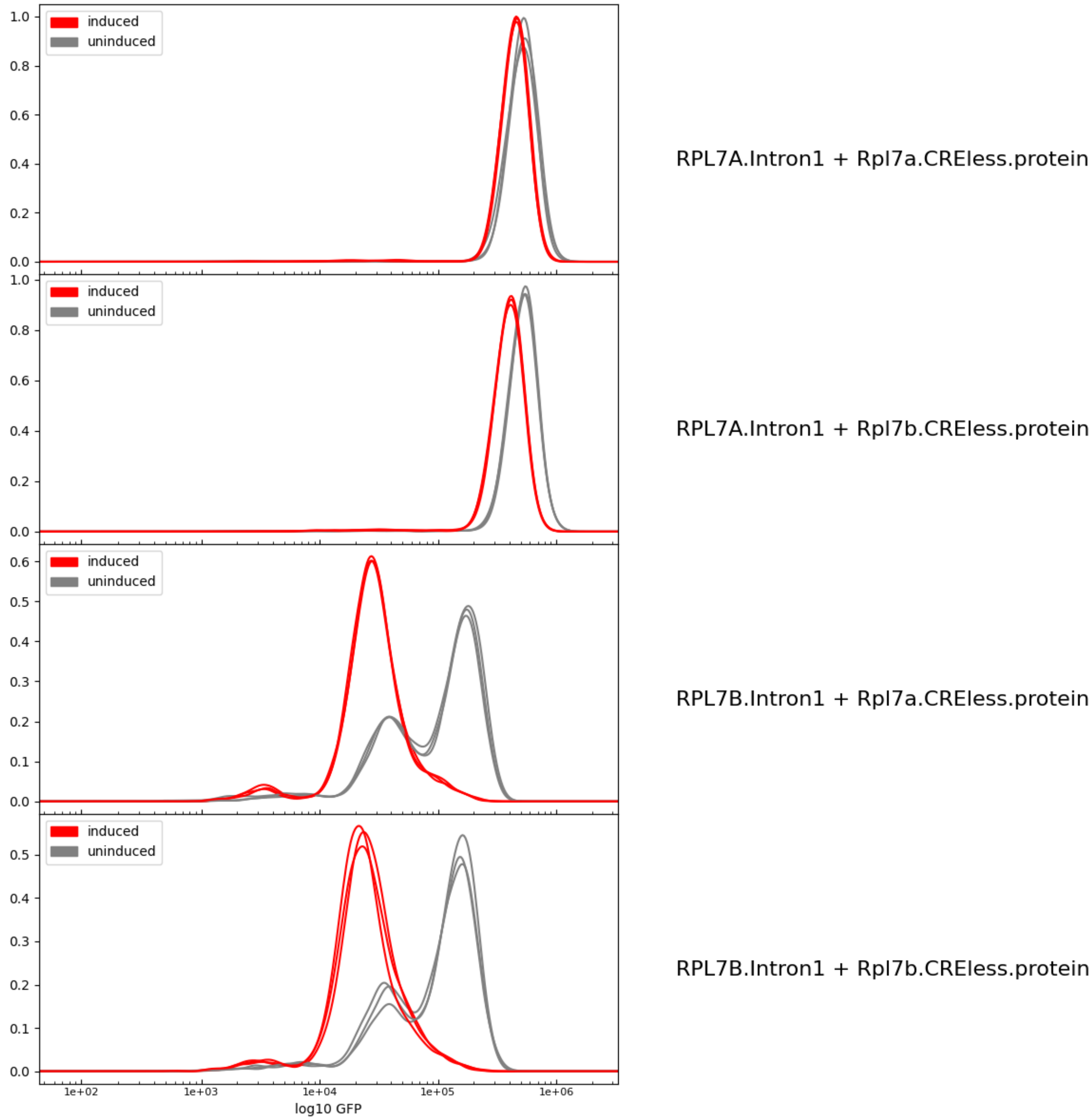

Figure S4 Alignment of intron 1 from five yeast species, as determined by PhastCon in the UCSC browser

```
RPL7B 1st Exon
sacCer ATGTCCACTGA
sacPar ATGTCCGCTCA
sacMik ATGTCTTCTCA
sacKud ATGTCTTCTCA
sacBay ATGTCTTCTCA

RPL7B 1st Intron
sacCer GTATGTCAAAAACTACAAATACGACCTTATTTGGTAACTAGTTTGTTG
sacPar GTATGTCAAAAA-TAAGAACAGGACCTTATTTGATAACTAATTTGTTA
sacMik GTATGTCAAGAATCAAGAATGCAGCCTCA-TAAGCAGCTAATCC-TTA
sacKud GTATGTCAAGAACTACAACTAGGACCTCATCTGGTAACTAATTG-CTA
sacBay GTATGTCAGAAACTTTGAACGTGGCC-CATCGGGTAACTAATTA-ATA

RPL7B Splicing Enhancer Left
sacCer TCGGTTACATCTGAAT-TTGAAGTACCCA
sacPar TCGGTTACATTTGAAT-TTGAAGTACCCA
sacMik TCGGTTACATTAGAGT-TTAAAGTGTCCA
sacKud TCGGTTACATTGGAATATTGAAATACCAA
sacBay TCGGTTACATTAGAATACTGAAATATCCA

RPL7B 1st Intron continued
sacCer AAT-TGATAATACTTCA-GGAAAAGTTAACGC-----ACATTCAA----------A-ACGTGTTTT-TTTAC
sacPar AAT-TGCTAAAACTTCA--GAAGGACGAATGCACATTACATTCAA----------ACAACTGTTTTAATTAT
sacMik CAT-CGAGAATACTTTA--GGGAAATAAATGCATAATGCATTCAA----------ACAGCAGACCA-TCAAG
sacKud -------------------GAGAGCCAAATGG-----ACATTTAATATG------ACAATTGGATT-AACAG
sacBay GATGTATTGATATTCCAGGGAAAGGCGATTGGACAATAAAATTAGTGGGGAAAAAACAACTGATTGATTCAA

RPL7B 1st Intron continued
sacCer AATAACCTATGGTGATCGAACCTAATTATAAAAAGAAGCAC-TTGAAGTAGTTTATCTTCTTCAAGTACCTT
sacPar TCTAATCTGTGGTAGTTGGGCTTAATC---AAAAGAGATAC-TTAAGTTAGTTTGTCTACTT-GAGTATTTT
sacMik GCTTGTTTGTGATTATCGAACTCAATA---AAAAGAGTAACACTGAGGCAGTTTG----CTT-CAGTGTTTT
sacKud TCTGGTGCACGTTAACTGGACTTAAT----AAGCAAGTTAC-TTGAGGCAGTTCATGTACTC-AAGTTTTTT
sacBay TTCAGTTTGTTCTGACTGATTTTTATA---AACCAAGTTAT-TTGAGATAGTACATCTTCTC--AATATCTT

RPL7B 1st Intron continued
sacCer TCGTAGGGGATTTTCATTTTTAATGAACAAAGGCATTGTTTGTTAGTATCTATTACTTGTAATACACCGATA
sacPar TCGTAGGGGAATTTCATTTTTAATGAACAAAGACATTGTTTGTTAATATCTATTACTTGTAATACACCAATA
sacMik TCGTAGGGGATTTTCATTTTTTATGAACAAAGGCATTGTTTGTTAATATCTATTACTTGCGATACACCAATA
sacKud TCGTAGGGGATTTTCATTTTT-ATGATCAAAGACACTGTTTGTTAATATCTATTGCTTGTAATACACCAATA
sacBay TCGTAGGGGATTTTCATTTTT-ATGAACAAAGGCATTGTTTGTTAATATCTATTACTTG--ATACACCATTA

RPL7B 1st Intron continued
sacCer ATTTCATTGATAGAT-ATTTCCAGCATGTCTCTGCATCTCCCTTCCATCGAATGAA
sacPar ATTTCATTAATAGAT-ATTTCCGGCGTGTCTCTGCATCTCCCTTCCATCGAATGAA
sacMik ATTTCACCAATAGAT-ATTTCCGGCGTGTCTCTGCATCTCCCTTCCATCGAATGAA
sacKud ATTTCATTAATAGAT-ATTTCCGGCATGTCTCTGCAACTCCCTTCCATCGAATGAA
sacBay ATTTCATCAATAGATCATTTCCAACATGTCTCTGCATCTCCCTTCCATCGAATGAA

RPL7B Splicing Enhancer Right
sacCer AAGTGGTGTTTTCAATTCGATGTAACTGA
sacPar AAGTGGTGTTTTCAATTCGATGTAACTGA
sacMik AAGTGGTGTTTTCAATTCGATGTAACTGA
sacKud AAGTGGTGTTTTCAATTCGATGTAACTGA
sacBay AAGTGGTGTTTTCAATTCGATGTAACTGC

RPL7B 1st Intron continued to end
sacCer ATCTTTGTTCTTAATATGACTTCTTTGCTAACATTTTTTTCATTTTTGAATAG
sacPar ATTTTTGTTCCTTATAGGACTTCTTTGCTAACA-TTTTTTCATTTTTGAATAG
```

sacMik ATTTCTGTTCCTTATAGAACACTTATGCTAACA----TTTCTTTTTTGAATAG
sacKud ATCTTTGATCCGTATAGGACCTCTTTGCTAAC--TTTTTTTTTTATTGAATAG
sacBay ATCTTTGATTTGTATTGAACTTTTATGCTAACA-TTTTTTTTTT-----TAG

RPL7B 2nd Exon
sacCer AAAAATCTTGACTCCTGAATCTCAATTGAAGAAGACTAAAGCTCAACAAAAGACTGCAGAACAAATTGCTGC
sacPar AAAAATCTTGACTCCCGAATCTCAATTGAAGAAGACTAAAGCTCAACAAAAGACTGCAGAACAAATTTCTGC
sacMik AAAAATCTTGACTCCTGAATCTCAGTTGAAAAAGAATAAAGCTCAACAGAAGACTGCAGAACAAACTGCAGC
sacKud AAAAATCTTGACTCCCGAATCTCAGTTGAAAAAAGCCAAGGCTCAACAAAAGAGTGCAGAACAAACTGCCGC
sacBay GAAAATCTTGACTCCTGAATCCCAATTGAAAAAGGCCAAGGCCCAACAAAAGACCGCTGAGCAAACTGCCGC

RPL7B 2nd Exon
sacCer AGAGAGAGCTGCCCGTAAAGCC
sacPar AGAAAGAGCTGCCCGTAAAGCC
sacMik AGAAAGAGCTGTCCGTAAAGCT
sacKud CGAAAGAGCTGATCGCAAAGCT
sacBay CGAAAGAATTGCTCGTAAAGCT

RPL7B 2nd Intron
sacCer GTATGTTCATTTACCATGTTTGAAAGATATTATATCATTCCTTTTACAGTGAGTTCACAAAAATA-TAATAC
sacPar GTATGTTCA-TTACCAAGTTTGAAAGATAT--TATCATTCCTTTTACAGTGAGTTCACAGAAATATTAATAT
sacMik GTATGTTAATTTGCCAAGATTGAAAGATAG--TATCAT---TTTTACAGTGAGCTCAC-GAAATA-TAATAC
sacKud GTATGTTCATTTGCCACGTTCAAAAAATAT--TATCATTCCTTTTATAGTGAGTTCAC--AAATA-TAATAT
sacBay GTATGTTGATTCATTGCACTTAAGAAATAT--TATCATTCGTTTTAAAGTGAGTTCAC--AAATA-TAATAT

RPL7B 2nd Intron
sacCer TTTGTTAAAGAGA---TCTTTTCTCGGTTTTACATTTCCTTGGCATTTGTTAGTCGCGAACAATTTT-----
sacPar TTTACTACTGTAT---TCTTCCTTCGGTTTTACATTTACTTGACATATGTTAAT-GCGAACGATTTT--TTT
sacMik TTTGTTAACAAAA-------CTCTCCGTTTTACATTTACTGGGCATGTGTTAAC-GCTAACGAATTT---CT
sacKud TTTGTCCAGAAACC-TTTTTTCTTCAGTTTCACATACACTTCGCATATGCTAGT-ACGGATGAACCTTCTTC
sacBay TTTACCAAGAAGACTTTTTTCCTTCAGTTTTACATTTACTCGGCATATGTTAAT-GCGGATGAATTC---CC

RPL7B 2nd Intron
sacCer -----------------CACTTTTTGCAACGTTTTTTTTCTTTCTTAATGATGAAAACTATTCCTTATTCTC
sacPar CGTTGACAATTTTC---CACTTTTTGCTACG----TCTTATTTTTCAATGATGAAAACTA-ACCTTATTCTC
sacMik CGTTGACAATTTT----CACTTTTTTCTACG----TCTTATTTTTC-ATGATGAAAACCATTCCATATTCTC
sacKud TGCTAATAGTTTTCCCATTTTTTTTGTTAAT----TCTTACTTTTT-ATGATGAAAACCATTCCTTTTTCTC
sacBay TATTAATAGTTTTCAACCATTTTTTGTT-CA----TCTTATTTTTT-GTGATGAAAACCATTCCTTTTTCTC

RPL7B 2nd Intron
sacCer GACTAGTCTTTGACAATGCTGTCGTTTAATCACCATCTTTCGGCTGACTAGTAAAGAAAATGCAAGAGT--G
sacPar GACTAGTCTTTGACAATGCTGTCGTTTAATCACCATCTTTCGGCTGACTAGTAAAGAAAG-ACAAAAAA--G
sacMik GACTAGTCTTTGACAATGCTGTCGTTTAATCACCATCTTTCGACTGACTAGTAAATAAAG-ACAAGGAA--A
sacKud GACTAGTCTTTGACAATGCTGTCGTTTAATCACCATCTTTCGACTGACTATTTAAGAAAG-GCAAAAAAGTG
sacBay GACTAGTCTTTGACAATGCTGTCGTTTAATCACCATCTTTCGGCTGACCAATTAAGAA---ACAAAAAA-TG

RPL7B 2nd Intron
sacCer AGAATATGCCAAGAATCTGCAAGATTGAGTT---AAGTTTTC--TTAGAAAGTTTTATCGTGCATAT-----
sacPar TGAATATGCCAAGAATTTGCAAGCCCAAGCT---AAGTTTTATGTTAGGAAATATTATTGTGCATATCTCCC
sacMik TGAGAATGCCAAGAAAATGCAAAACTAAGCT---AAGTTTTATGTTAAAATGTATTTATGTCTACCT----C
sacKud TGAATATGCTGAGTGTATGCCAGACTACTCTAAAAAGTTTTATGC----AAATATTTATATTTTTAA-----
sacBay TGAAAATGCTAAAGAAATGTAAAACCAAGTTTAAGAGTTTTG---TAAAATCTATT-CCGTGCAACT-----

RPL7B 2nd Intron
sacCer TTTTCACTAAATTGGAATGACGTTA----------AAACCAAAAGTACTGATCTTACTAA----CATTAATC
sacPar TCTTCACTGATATGGAATGATACTA---------TAAACTAAATGTATTTACCTTACTAA----CATCAATC
sacMik CCTGCACTGATATGAAATGATTTTC---------CTCGTCAGAAGCATTAGTTTTACTAA----CATGAATC
sacKud -CTCCATTGACATGAAATGACATCA---------TGAACCACAAAA---TGTTTTACTAA----CATGAATT
sacBay -TACTACAGACGTAGTGTGATATTATGTGAACACTTGAACAAAAAATCTTTTTTTACTAACAAGCATATATT

RPL7B 2nd Intron
sacCer AAA-AATTCTTGATGAATATTATTTTAG
sacPar AAACGATTCTTGAT-AACATTATTCTAG

sacMik ATAAAATTCTTGAT-ATTTTTTTAATAG
sacKud AAACAAATCTCAAT-ATAATGTTCTTAG
sacBay ATTAAATTCTTAAT-ATCATTTTCTTAG

RPL7B 3rd Exon
sacCer GCTAACAAGGAAAAAAGAGCTATTATTTTGGAAAGAAACGCCGCTTACCAAAAGGAATACGAAACTGCTGAA
sacPar GCTAACAAGGAAAAAAGAGCTATCATTTTGGAAAGAAACGCTGCTTACCAAAAGGAATACGAAACTGCTGAA
sacMik GCCAACAAGGAAAAGAGAGCTATCATTTTGGAAAGAAACTCTGCTTACCAAAAGGAATACGAAACTGCTGAA
sacKud GCTAATAAGGAAAAAAGAGCTATCATTTTGGAAAGAAACGCCGCTTACCAAAACGAATACGAAGCTGCTGAA
sacBay GCTAACAAAGAAAAGAGAGCTATCATTTTGGAAAGAAACGCTGCTTACCAAAAGGAATACGAAACTGCTGAA

RPL7B 3rd Exon
sacCer AGAAACATCATTCAAGCTAAGCGTGATGCCAAGGCTGCTGGTTCCTACTACGTCGAAGCTCAACACAAGTTG
sacPar AGAGAAATCATTCAAGCCAAGCGTGATGCCAAGGCTACTGGTTCCTACTACGTCGAAGCTCAACACAAGTTG
sacMik AGAGACATCATCCAAGCCAAGCGTGATGCAAAGGCTGCTGGTTCCTACTACGTCGAAGCTCAACACAAGTTG
sacKud AGAGACATCATTCAAGCCAAACGTGATGCGAAGGCCGCTGGTTCTTACTACATTGAAGCTCAACAAAAGTTG
sacBay AGAGACATCATCCAAGCCAAGCGTGATGCTAAGGCTACTGGTTCCTACTACGTCGAAGCTCAACGTAAGTTG

RPL7B 3rd Exon
sacCer GTCTTCGTTGTCAGAATCAAGGGTATTAACAAGATTCCACCTAAGCCAAGAAAGGTTCTACAATTGCTAAGA
sacPar GTCTTTGTCGTTAGAATCAAGGGTATTAACAAGATCCCACCTAAGCCAAGAAAGGTTTTGCAATTGCTAAGA
sacMik GTCTTTGTTGTCAGAATCAAGGGTATCAACAAGATCCCACCTAAGCCAAGAAAGGTCTTGCAATTGTTAAGA
sacKud GTCTTCGTTGTCAGAATCAAGGGTATCAACAAGATTCCACCTAAACCAAGAAAGGTTCTACAATTGCTAAGG
sacBay GTCTTCGTTGTTAGAATCAAGGGTATCAACAAGATCCCACCTAAGCCAAGAAAGGTTCTACAATTGCTAAGA

RPL7B 3rd Exon
sacCer TTGACAAGAATCAACTCTGGTACATTCGTCAAAGTTACCAAGGCTACTTTGGAACTATTGAAGTTGATTGAA
sacPar TTGACCAGAATCAACTCCGGTACTTTCGTCAAAGTTACTAAGGCTACCTTGGAACTATTGAAGTTGATTGAG
sacMik TTGACCAGAATCAACTCTGGTACTTTCGTGAAAGTTACTAAGGCTACTTTGGAATTGTTGAAGTTGATCGAA
sacKud TTGACCAGAATCAACTCTGGTACTTTCGTCAAAGTCACTAAAGCTACCTTGGAATTGTTGAAGTTGATTGAA
sacBay TTGACCAGAATCAACTCCGGTACCTTCGTCAAGGTTACCAAGGCCACTTTGGAATTGTTGAAGTTGATCGAA

RPL7B 3rd Exon
sacCer CCATACGTTGCTTACGGTTACCCATCCTACTCTACTATTAGACAATTGGTCTACAAGAGAGGTTTCGGTAAG
sacPar CCATACGTTGCTTATGGTTACCCATCATACTCTACTATCAGGCAATTAGTCTACAAGAGAGGTTTCGGTAAG
sacMik CCATACGTTGCCTACGGTTACCCATCCTACTCTACTATCAGACAATTAATCTACAAGAGAGGTTTCGGTAAG
sacKud CCATACGTAGCTTACGGTTACCCATCTTACTCTACCATCAGACAGTTAGTCTACAAGAGGGGTTTCGGTAAG
sacBay CCATATGTTGCTTACGGTTACCCATCTTACTCTACCATCAGACAATTAGTCTACAAGAGAGGTTTCGGTAAG

RPL7B 3rd Exon
sacCer ATCAACAAGCAAAGAGTTCCATTGTCCGACAATGCTATCATCGAAGCCAACTTGGGTAAGTATGGTATCTTG
sacPar ATCAACAAGCAAAGAATTCCTTTGTCCGACAACGCTATCATCGAAGCCAACTTGGGTAAGTATGGTATCTTA
sacMik ATCAACAAGCAAAGAATTCCTTTGTCCGACAACGCTATCATCGAAGCTAATTTGGGCCAATACGGTATCTTG
sacKud ATCAACAAGCAAAGAGTTCCATTGTCGGACAACGCTATCATTGAAGCTAACTTAGGTAAGTACGGCATCTTG
sacBay ATCAACAAGCAAAGAGTCCCATTGTCCGACAACGCTATCATCGAAGCTAACTTGGGTAAGTACGGAATCTTG

RPL7B 3rd Exon
sacCer TCCATTGACGATTTGATTCACGAAATCATCACTGTTGGTCCACACTTCAAGCAAGCTAACAACTTTTTGTGG
sacPar TCCATTGACGATTTGATTCATGAGATCATTACTGTCGGCCCACACTTCAAGCAAGCCAACAACTTCTTGTGG
sacMik TCCATAGACGATTTGATTCATGAAATCATAACTGTCGGTCCACACTTCAAACAAGCTAACAACTTCCTGTGG
sacKud TCTATTGACGATTTGATTCACGAAATTATCACTGTCGGTCCACATTTCAAGCAAGTCAACAATTTCTTGTGG
sacBay TCCATTGACGATTTGATCCACGAAATCAAAACCGTTGGCCCACACTTCAAGCAAGCCAACAACTTTTTGTGG

RPL7B 3rd Exon
sacCer CCATTCAAGTTGTCCAACCCATCTGGTGGTTGGGGTGTCCCAAGAAAGTTCAAGCATTTCATCCAAGGTGGT
sacPar CCATTCAAGTTGTCCAACCCATCTGGTGGTTGGGGTGTCCCAAGAAAGTTCAAGCACTTTATCCAAGGTGGT
sacMik CCATTCAAATTGTCCAACCCATCCGGTGGTTGGGGTGTCCCAAGGAAGTTTAAGCACTTTATCCAAGGTGGT
sacKud CCATTCAAGTTGTCCAACCCATCTGGTGGTTGGGGCGTCCCAAGAAAGTTCAAGCACTTTATCCAAGGTGGT
sacBay CCATTCAAGTTGTCCAACCCATCCGGCGGTTGGGGTGTCCCAAGAAAGTTCAAGCACTTTGTCCAAGGTGGT

RPL7B 3rd Exon
sacCer TCTTTCGGTAACCGTGAAGAATTCATCAATAAATTGGTTAAGGCTATGAACTAACAT----TATTCCGTGTG
sacPar TCTTTCGGTAACCGTGAAGAATTCATCAACAAATTGGTTAAGGCCATGAACTAGGGT----TATGTCGTATG

```
sacMik TCCTTCGGTAACCGTGAGGAATTTATCAATAAGTTGATCAAGGCTATGAACTAAGAA----CATGTCTCATG
sacKud TCTTTCGGTAACCGTGAAGAATTCATTAACAAGTTGATCAAGTCTATGAACTAAAATGGGCGATGTCGTATG
sacBay TCTTTCGGTAACCGTGAAGAATTCATCAACAAGTTGATCAAGTCCATGAACTAAGTA-GCTGACGCCGGAAG

RPL7B 3rd Exon
sacCer GCAA-TAA-TCTCAATGTATAATAAATAATATTTCTCTT-CATATA----------TATGACCGGAC-----
sacPar GCAA-CAA-TCTCGATGTATAATAAATAATATTTCTCTT-CATATACGCATATATCTATGACCGGGC-----
sacMik GCAA-TGT-ACTTGATGTATAATAAACGTAATTTCTCCTACATATAATTATATATATGTATCTAAAC-----
sacKud ACAAGTTT-TCTCAATGTATATTAAACAAGATTTATTT--CGTATACCAACATATTTGTATATAATCACAAA
sacBay GCGA-TATCTCTTAATGTATAAT-------ATTTCATT--CATATA------------TAATCAATCACACA

RPL7B 3rd Exon
sacCer --TCGTAATAGAATAATTGACTGGAACAATAGCGC--ATATTGCTTTGCTTCTATACGTAGTTGAGT-TTTC
sacPar --TCCTAATAGAATAACTGACTGGAATAATAGTGC--AAACTCCTTTATTTCTATATGTAGTTGAGT-TTTT
sacMik --TCTTAATAGAATAATTGACTGGGTCAAT--TGC--TAAGGGCTTTGTTCCCCTACATAGTTGAGT-TTTT
sacKud CAACGTAATAGAACAATAGGCTGGGTAAAAAGCAC--GGAAAGGTTTGCTCCTATTTATAGTTGAGTCCTTT
sacBay TAACGTAATAAAGCAATTGACTGGCTCAAAAATGCGAAGAGGGCTTTTCTTCCGTTTGTAATCGAGCCCTGT

RPL7B 3rd Exon
sacCer A
sacPar A
sacMik T
sacKud G
sacBay G
```

Supplemental Table S1

Primers used for assessing binding by Rpl7 to intron 1

Primer Name	Sequence (5′ → 3′)	Target
RIP-GFP_cDNA	GTAAGTAGCATCACCTTCACCTTCACCGGAGACAG	GFP
RPL7A_cDNA	CAGCTCTTTCAGCAGCGACTTGTTCAGC	RPL7A
RPL7B_pull_cDNA	CTTGATTCTGACAACGAAGACCAAC	RPL7B
T7_hooks_structure_F	TAATACGACTCACTATAGGGTGAAGTAGTTTATCTTCTTCAAGTACC	GFP & RPL7B
T7_hooks_structure_R	CAGGAGTCAAGATTTTTCTATTCAAAAATG	GFP & RPL7B
RPL7A_Intron_F	TGGCCGCTGAGTATGTATACG	RPL7A
RPL7A_Intron_R	CCTTAGACTTCTTCAACTGAGATTCTGG	RPL7A
